# Skeletal Muscle Activity and the Fate of Myonuclei 

**Published:** 2010-07

**Authors:** B.S. Shenkman, O.V. Turtikova, T.L. Nemirovskaya, A.I. Grigoriev

**Affiliations:** Institute for Biomedical Problems, Russian Academy of Sciences

**Keywords:** skeletal muscle, myonuclei apoptosis, physical training, working hypertrophy, satellite cells, growth factors, gravitational unloading, muscle stretch

## Abstract

Abstract Adult skeletal muscle fiber is a symplast multinuclear structure developed in ontogenesis by the fusion of the myoblasts (muscle progenitor cells). The nuclei of a muscle fiber (myonuclei) are those located at the periphery of fiber in the space between myofibrils and sarcolemma. In theory, a mass change in skeletal muscle during exercise or unloading may be associated with the altered myonuclear number, ratio of the transcription, and translation and proteolysis rates. Here we review the literature data related to the phenomenology and hypothetical mechanisms of the myonuclear number alterations during enhanced or reduced muscle contractile activity. In many cases (during severe muscle and systemic diseases and gravitational unloading), muscle atrophy is accompanied by a reduction in the amount of myonuclei. Such reduction is usually explained by the development of myonuclear apoptosis. A myonuclear number increase may be provided only by the satellite cell nuclei incorporation via cell fusion with the adjacent myofiber. It is believed that it is these cells which supply fiber with additional nuclei, providing postnatal growth, work hypertrophy, and repair processes. Here we discuss the possible mechanisms controlling satellite cell proliferation during exercise, functional unloading, and passive stretch.

## INTRODUCTION

Skeletal muscle is the most flexible structure in mammalian organisms. High muscle activity and load often lead to an increase in the transverse size (thickness) of the muscle, myofibrils volume, and contractile properties (strength and power). The stable pattern of gene expression underlies such transformation.

A chronic decrease in the functional load on the postural muscles, primarily soleus, under a prolonged change in the action of gravity forces (bed rest, support elimination from all or only hind limbs, or weightlessness) – so–called gravitational unloading – deeply transforms all the structural and functional muscle–tissue machinery [1–3]. One of the most important consequences of muscle transformation under hypogravity is the decrease in the contractile properties (power and working capacity), stiffness of muscle and myofibers, and a significant decline in myofibril and nuclei number, as well as in fiber size (atrophy). It also leads to an overgrowth of the connective tissue and extracellular structures and a shifting of the phenotype of myosin heavy chains towards an increase in the expression of the fast isoforms of the myosin heavy chains. The data obtained in the last years showed that the gravity–dependent transformation of soleus fibers is based on a stable directional change in the expression of a number of genes and the generation of a new integral (the so–called atrophic) expression pattern. 

 Adult muscle fiber is a symplast ,a multinuclear structure, developed in ontogenesis by the fusion of myoblasts (muscle progenitor cells). Nuclei are located at the periphery of muscle fiber in the space between myofibrils and the cell membrane (sarcolemma). Muscles contain also the nuclei of fibroblasts, endothelial cells, and precursor cells (satellite cells). Thus, in the literature, muscle fiber nuclei are usually called myonuclei. In theory, as a result of disuse or overload, the skeletal muscle mass can change, because of a change in the number of myonuclei or alterations in the rates of transcription, translation, and proteolysis. In this work we review data accumulated in the literature concerning the phenomenology and possible mechanisms of changes in the quantity of muscle fiber nuclei during increased or decreased contractile activity.

The muscle fiber nuclei are postmitotic and cannot divide. Myonuclei quantity is extremely important, since it determines the content of DNA for gene transcription [4]. The interaction between the fiber size and myonuclei number was taken as the basis in the myonuclear domain concept offered by Cheek *et al * . [5]. Myonuclear domain is the volume of muscle fiber cytoplasm regulated by the expression of the genes of one nucleus. The term “myonuclear domain” is quite convenient for describing the mechanisms of muscle plasticity, though it is nominal, and the protein distribution inside muscle fiber depends on many variable parameters. A lot of studies have analyzed the cross–sectional area per one myonucleus, instead of the domain.

To reveal the myonuclei, DNA–specific dyes are used. The main problem for researchers of the nuclear pool of muscle fiber face is that, in an analysis of the muscle transverse sections without special techniques, it is impossible to distinguish the nuclei located on different sides of the muscle fiber boundary. To solve this problem, different approaches are used; in particular, the double labeling of nuclei and specific proteins of the subsarcolemmal layer, such as dystrophin [6]. Many authors have analyzed the nuclear composition of the isolated muscle fiber [7, 8]. To study an isolated fiber, which is a volumetric structure, a confocal laser microscope should be used. This approach has evident advantages: all of the myofiber nuclei pool can be analyzed (not only the nuclei observed at the cross section); also, the nuclei density distribution along the muscle fiber and its elementary unit, sarcomere, can be traced. However, the number of the fibers is limited in this case by 20–30 fibers per one biological sample.

Allen * et al * . have offered a hypothesis of myonuclear domain constancy during the size changes of the muscle fibers (atrophy and hypertrophy) [4]. The authors showed that the myonuclear domain size remains stable during the acute stage of hypertrophy. A proportional increase in the myonuclei quantity and cytoplasm volume was observed on a model of functional hypertrophy caused by the removal of synergistic muscles [9]. The same authors showed the variability of the myonuclear domain size during a chronic increase or decrease in loads in dogs [10] and under atrophy in rats [7]. Thus, the hypothesis of the myonuclear domain constancy turned out to be indefensible, and it has been further disproved in numerous studies of disuse and training [11–13, 8]. The myonuclear domain has been found to change throughout an animal’s lifetime [14–16]. Recent studies by Italian authors have proved the possibility of hypertrophy development without new myonuclei incorporation; i.e., without a myonuclear domain increase under hypertrophy [11].

## Myonuclear number reduction

In a number of cases (during severe muscle and systemic diseases and under gravitational unloading), muscle atrophy is accompanied by a decrease in the myonuclei number per myofiber, along with a corresponding development of apoptotic processes in the myonuclei. Such a reduction in the nuclei number was observed in cosmonauts’ quadriceps [17] and rat soleus after space flight [10, 12], under simulated unloading in rats using the so–called hindlimb suspension technique [18, 19], and during soleus immobilization. Myonuclei loss is most intensive in slow fibers [19]. Studies of single fibers have demonstrated a decrease in the myonuclear domain size under disuse in rat soleus, but not in plantaris [12]. The myonuclear domain of rhesus monkeys also tends to decrease after 14 days in space flight [20]. Wang * et al* . showed a reduction of the cross sectional area, myonuclei number (25%), and nuclear domain size of soleus fibers after 16 days of rat hindlimb suspension [21].

Myonuclear number reduction is explained by the nuclei apoptosis in muscle fibers. Apoptotic processes in muscle fibers develop differently than those in other cell types. The changes in contractile activity lead to a weak manifestation of ultrastructural nuclei destruction. At the same time, DNA breaks in the nuclei are accompanied by a number of mitochondrial and extramitochondrial events which are supposed to be components of the interdependent signaling pathways, which cause the apoptotic processes.

Apoptotic nuclei have been observed in the muscle fibers of patients with Duchenne dystrophy (and in its biological model, mdx mice) [22], in fibers affected by chronic heart failure, the development of amyotrophic lateral sclerosis, and in some other cases. Myonuclei apoptosis was also observed after the application of a specific physical load (so–called eccentric exercise) [23]. In this case, muscle fiber strain develops when the fibers are stretched. Such muscle contraction causes numerous destructive changes in the cytoskeletal proteins and sarcolemma. In 1997, Allen *et al * . first reported the presence of apoptotic nuclei during rat hindlimb suspension [18]. The maximum number of apoptotic nuclei was observed in the soleus fibers (according to the TUNEL staining, revealing the DNA breaks) in the 2nd day of soleus disuse [24].

The same data were obtained in experiments on mice, where the maximum apoptosis–inducing factor (AIF) and p53 expression after 24 h of disuse were determined [25]. This was preceded by a marked increase in caspase–3 and caspase–8 after 12 h of suspension. An increased concentration of Bcl–2 was found as early as after 6 h of disuse. During hindlimb suspension for over 24 h, the observed apoptotic manifestations decreased. Soleus immobilization revealed similar dynamics [24]. Seven days of reloading after hindlimb suspension were enough to eliminate apoptosis [26]. Some authors were unable to find caspase cascade activation in soleus under hindlimb unloading or spinal isolation [27], but they observed endonuclease G translocation to the nucleus. Endonuclease G is the mitochondrial enzyme degrading nuclear DNA. Recently, a group of authors suspended animals under decreased temperature, which is supposed to slow down the mitochondria–dependent processes. In this case, apoptotic nuclei and significant caspase activation were also observed [28].

As was mentioned previously, a single exercise bout caused different apoptotic manifestations (DNA fragmentation, increased caspases activity, etc.); however, regular physical training not only decreased such apoptosis manifestations, but it also had an antiapoptotic effect that eliminated the nuclear changes that take place when muscle activity is reduced [18].

Unlike the other cell structures, in a skeletal muscle fiber apoptosis of the individual nuclei does not lead to immediate fiber death, though pathological consequences develop.

Recently, the Bruusgaard group [29] cast doubt on the complex of described observations of nuclear losses and apoptosis during atrophy. The authors studied mice transfected with the GFP–encoding plasmid. GFP was localized in the myonuclei, the quantity of which was analyzed under prolonged (14 days) denervation and disuse of extensor digitorum longus (caused by antagonists’ tenotomy). The authors observed a significant decrease in the cross–sectional area of the muscle fibers, but no myonuclei decline. Fixed apoptotic changes were found in the satellite and connective tissue cells only; they were not revealed in muscle fibers. The same results were observed during the detraining of the Japanese quail wing: all the nuclei with apoptosis features were labeled with bromdeoxyuridine (BrdU), the DNA synthesis indicator, which revealed the satellite cell nuclei [30]. At the same time, the conclusion made by Bruusgaard and Gundersen [29] was based on the denervation experiment and a study of the blockade of nerve impulse conduction to muscles predominantly with the fast fibers (those fibers undergo apoptosis less; see above). The authors dispelled any doubt about the data testifying to apoptosis and myonuclei number reduction using an animal microgravity model of antiorthostatic hindlimb suspension (and, consequently, the results of analogous experiments on volunteers; see above). They assumed that, in this case, systemic manifestations of gravitational unloading favored myonuclei apoptosis. Unfortunately, this hypothesis has no experimental support.

An increase in myonuclei in adult muscle fiber was observed during a power exercise, experimental working hypertrophy (during synergistic muscles dissection), and postatrophic reloading [26, 31–33]. The new nuclei in a fiber can be provided only by the fusion of satellite cells with the muscle fiber. The satellite cells are supposed to provide new nuclei for the muscle fiber during the postnatal period and for the local regeneration of the injured muscle fibers [34].

## Precursor cells in skeletal muscle. The markers of the myosatellite cells.

Satellite cells in a skeletal muscle are small mononucleate resting cells (remaining in the G_o_ phase of the cell cycle) which proliferate and fuse with muscle fibers when activated, being an essential source of myonuclei during postembryonic development under tissue hypertrophy and recovery [12]. They can also fuse with each other, forming new muscle fibers [16]. Satellites may be myoblasts resting in the muscle tissue. According to another opinion, satellites are also believed to derive from some endothelial precursors associated with the embryonic vascular system. They can rest in the skeletal muscle interstitial space and express CD 34 [35]. However, skeletal muscle myogenic precursors are more numerous than satellite cells, because of the migration or recruitment of the undifferentiated stem cells from other sources. The precursor cell population from skeletal muscle was shown to originate from virgin mesenchymal stem cells of bone marrow and differs from satellite cells. Unlike satellites, precursor cells of the side population express Sca–1 (stem cell antigen–1) and CD–45. Evidently, they take part in injured or transplanted muscle regeneration and potentially form myocytes and myosatellite cells [36, 37]. Myosatellites can be identified in a muscle by their location (between the sarcolemma and fiber basal lamina) and by the immunohistochemical identification of different proteins expressed by these cells at different stages of their cell cycles. Desmin, myf5, and MyoD were found in the activated proliferating satellite cells, which normally express the regulatory muscle factors, such as Pax–7 and c–Met. Myogenin and MRF4 synthesis is characteristic of the final stage of differentiation [38;]. c–Met, the HGF receptor, is expressed in skeletal muscle not only by satellites, but also by other myogenic precursor cells. Like resting cells, active and proliferating satellite cells usually synthesize such cell adhesion molecules as m–cadherin (Mcad) and NCAM (CD 56, Leu–19, neural cell adhesion molecule), which are located in the narrow space between the satellite cell and muscle fiber. NCAM is expressed in the activated satellite cells (myoblasts), in myotubes during muscle regeneration, and in neuromuscular junctions. Recent data showed that NCAM is the earliest marker of committed myoblasts; i.e., it determins their univocal transition from the proliferation to the differentiation phase [39].

The key molecule of the myogenic morphogenesis is Mcad. Using the combined labeling of Mcad, NCAM, laminin, desmin, and cell nuclei, Irintchev * et al * . [40] demonstrated that Mcad occurs in the satellite cells and myoblasts of normal and regenerating muscle. Simultaneous staining of the regenerating muscle with Mcad and BrdU led the authors to conclude that Mcad is expressed predominantly in mitotically inactive (resting) satellite cells. The myoblast fusion suppressed Mcad expression. NCAM and Mcad simultaneous expression is often observed in the muscles with an innervation failure [40]. As was shown later [41], skeletal muscle hypertrophy caused by overloading and the number of myosatellites expressing Mcad at the early stages of stimulus application increased, while at the later stages the number of cells positively stained against Mcad and NCAM rose. Thus, the Mcad staining was found in the resting myosatellites similarly as in the proliferation and differentiation stages.

## Myosatellite cells under gravitational unloading

Three days of hindlimb unloading lead to an irreversible transformation in the muscles of young rats. Therefore, the satellite cell number and their proliferative potential (according to the BrdU incorporation data) declined in soleus in the same way as in extensor digitorum longus. In this case, the program of muscle fiber development in growing animals can change irreversibly, leading to a failure of the myonuclei number increase even after reloading [42, 43]. Satellite cell mitotic activity decreased after 24 h of disuse and completely deceased 3–5 days later. The most pronounced decline was observed in soleus. Morphological atrophy features were revealed 48 h later [43]. The increase in the proliferative processes in mice gastrocnemius appeared after one week of hindlimb suspension [44]. The quantity of the resting and mitotically active satellites in muscle fiber fell by 57% when compared to the control group [29]. In the other work by the same authors, 3 months of unloading caused no decrease in the satellite cell quantity or muscle fiber length in young animals, but it led to an apoptosis–independent decrease in the satellite cell and myonuclei contents and a decrease in the satellite mitotic activity [45]. However, Ferreira * et al * . [44] observed unexpected proliferation reinforcement in mice gastrocnemius after one week of suspension.

## Possible ways of activating myosatellites

Myosatellites are supposed to rest when skeletal muscle is not active. Their activation provides muscle mass maintenance, hypertrophy development, or the recovery of injured muscle. Myosatellite activation can also be caused by strengthening exercise [46, 47].

**Fig. 1:**
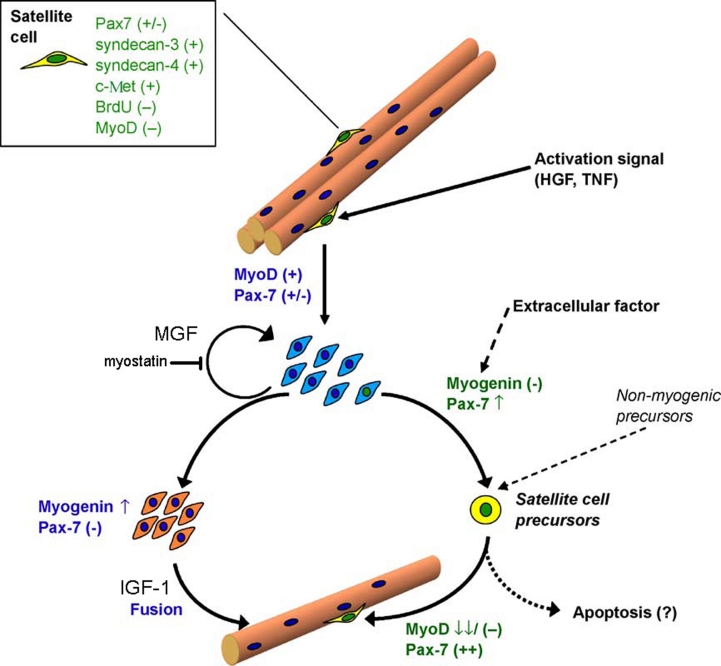
The hypothetical role of IGF-1 and MGF in satellite cell physiology. Modified scheme of Olguin and Olwin, 2004 [54].

A significant increase in the total number of satellites was shown on several models of compensatory hypertrophy in animals, after eccentric exercise in humans [13, 48], and during muscle stretching [49]. Physical activity, such as resistive exercise or muscle functional overload (chronic stretch, synergistic muscle removal, and tenotomy), injures muscle tissue [50], stimulating muscle regeneration. Muscle damage causes an inflammatory response. Therefore, in the injured area, the number of neutrophils and macrophages increases. Then, the inflammatory infiltrate is released by immune cells, or the injured fibers release the growth factors regulating the proliferation and differentiation of myosatellites. Cytokines ( IL–4, IL–6, IL–15, TNF– α etc. ) were shown to affect the satellite cells * in vitro * and during the regeneration of injured muscle [51]. The role of the fibroblast growth factor (FGF) in myosatellite activation has been shown previously [52]. The hepatocyte growth factor (HGF) is supposed to be the key regulator of satellite cells activity during regeneration [18, 53] (Fig. 1). HGF was established to stimulate satellite cell activation in culture and * in vivo* during muscle stretch. HGF release is induced by nitric oxide (NO) synthesis, and it is regulated by matrix metalloproteinases (MMPs) [55;]. HGF affects a satellite through its binding to the c–met receptor, stimulating further the signaling cascade, including the PI3K–Akt pathway, which stimulates cell survival and protection against apoptosis. The results of numerous experiments have shown the important role of the insulin–like growth factor in muscle hypertrophy development. In * in vivo * studies on animals, data were obtained demonstrating the role of an insulin–like growth factor (IGF) in the growth processes mediated by myosatellite activity [56, 57;]. IGF can stimulate myosatellite proliferation and differentiation in culture [58]. Myosatellite cells of mice with IGF–1 gene overexpression possess increased proliferative potential, which can be due to the activation of a PI3K–Akt signaling pathway and the decline of the blocker of the cyclin–dependent kinase–2 [59], which is a result of FOXO transcription factor inhibition [57]. This is why the IGF–1–activated signaling pathways, which stimulate translation, are also supposed to be activated in myosatellite cells [60]. However, IGF–1EA (the growth factor form expressed in liver cells and skeletal muscle fibers which releases into the blood flow) is not the only IGF–1 gene product.

Physical training or mechanical muscle damage results in IGF–1 gene splicing, leading to the appearance of a splice–variant called the mechano–growth factor (MGF) after 1–2 days. During splicing, the translational frame shift occurs, resulting in a C–terminal sequence change. This leads to the appearance of the so–called E–domain, which differs from the other IGF–1 splice–variant sequences [61]. This unique C–terminal peptide functions as an autocrine growth factor with a short half–life. One of the functions of the peptide is to increase the precursor–cell pool in skeletal muscle (satellite cells) by initiating stem–cell proliferation, however, without myogenic differentiation. After initial splicing leading to MGF formation, the IGF–1 gene product undergoes further splicing, generating the IGF–1EA isoform. IGF–1EA is supposed to stimulate myosatellite differentiation and fusion with muscle fiber [38, 49, 62]. However, according to Wozniak *et al * . [38], only HGF and NO have been proven to activate resting myosatellites. IGF, FGF, and other growth factors were shown to effectively stimulate proliferation and growth following satellite activation. Some other growth factors (in particular, FGF) can also activate satellite cell proliferation [52]. Myosatellite cell activation can be suppressed by myostatin, which is supposed to maintain myosatellites at rest [63]. However, the mechanisms inducing satellite cell activation, proliferation, and their fusion with injured or growing muscle fibers remain poorly understood.

## The role of precursor cells in muscle growth

Satellite cells possess high proliferative potential and are supposed to be important for skeletal muscle regeneration and hypertrophy. Different stimuli, such as functional overload during synergist removal, testosterone, clenbuterol, muscle stretch, and exercise can activate the satellites, stimulate their entry to the cell cycle and their proliferation in both fast and slow muscles. An increase in satellite proliferation was observed in the first days after stimulus application. Cramery * et al * . [46] and Kadi * et al * . [13, 31] showed that a series of intensive exercises stimulated an increase in the number of cells expressing NCAM. However, this and other studies did not show proliferating cells fusing with muscle fibers. The question of whether myosatellite nuclei incorporation into the fiber is necessary for muscle growth or mass maintenance remains unanswered. Different points of view exist. Many authors deny the necessity of incorporating myosatellite nuclei for muscle hypertrophy development [64], which has been proven by numerous studies with β 2–adrenoceptor agonists application, leading to muscle hypertropy without an increase in DNA or the myonuclear number. According to Kadi * et al * . [13], muscle fiber size can change moderately without the incorporation of new myonuclei. As was shown earlier, the myonuclear number is not normally the determining factor for muscle fiber size; the myonuclear domain size varies during an animal’s lifetime [16] and is unstable under muscle atrophy [65]. Despite the lack of dividing myosatellites after ionizing radiation treatment, Lowe [32] observed hypertrophy of the stretched slow anterior latissimus dorsi of the Japanese quail. Dupont–Versteegden * et al * . [66] showed that in spinalized animals, after myosatellite activation (during resistive exercise), the latter did not fuse with the muscle fibers. Thus, training did not promote the maintenance of the myonuclear number in the soleus of spinal animals. The number of activated myosatellites was higher than that of divided ones. The physiological role of activation of such a huge number of myosatellites without their incorporation into the growing muscle fibers is unclear. Recently, Italian researchers showed that proteinkinase B activation for 3 weeks caused muscle hypertrophy and a doubling of muscle weight, which was not accompanied by satellite activation or the incorporation of new nuclei [11].

The possibility of muscle fiber growth without incorporation of satellite cells, which is one of the ways protein synthesis intensifies, can be due to an increase in DNA matrices because of the incorporation of myosatellite nuclei into the fiber. The supporters of the concept of myonuclear domain constancy theorize that the initial stages of muscle growth are linked with transcription and translation intensification until the myonuclear domain reaches a definite threshold. However, it was established that moderate hypertrophy in human muscles can happen without additional genetic material [13]; from the point of view of the concept mentioned above, this can be explained by the existence of a hypertrophy threshold sensitive to the new nuclei incorporation. Thus, at later stages, the incorporation of new myosatellite nuclei is obligatory for maintaining muscle fiber hypertrophy and the nuclear domain size [9, 33, 64]. The necessity of myosatellites for muscle hypertrophy development was first shown by Rosenblatt * et al * . [9], who observed a decrease in hypertrophy under functional overload after averting satellite proliferation by γ –irradiation. The authors determined that satellite cell death under the irradiation and obviation of their nuclei’s incorporation into muscle fibers can completely neutralize the hypertrophy of rat extensor digitorum longus, soleus, and plantaris caused by the removal of synergistic muscles and physical training [67]. Mitchell and Pavlath [33] showed that, after rat hindlimb suspension and irradiation, the prevention of myosatellite proliferation muscle recovery was normal only at the stage where new myonuclei were not necessary, but then the process slowed. Kawano * et al * . [45] showed that, during 3 months of reloading young animals suspended for 3 months, the fiber cross–sectional area did not differ from that of the control animals, while the satellites and myonuclei number increased. The authors concluded that satellite cells were important for soleus growth processes [45]. As was shown earlier, the proliferation, differentiation, and fusion of myosatellites with muscle fibers are induced by growth factor IGF–1 [68]. In the muscle fiber culture, IGF–1 caused myosatellite fusion resulting in hypertrophy [62]. The IGF–1–stimulated hypertrophy was accompanied by an increase in DNA content in the muscle fibers and the appearance of new myonuclei [69]. The irradiation was shown to decrease the hypertrophy of the extensor digitorum longus caused by the intramuscular IGF–1 incorporation twice, inasmuch as in hypertropy induced by the incorporation of myosatellites nuclei into the muscle fibers [70]. These data showed that the loading–stimulated increase in the IGF–1 level can cause hypertrophy, particularly due to the stimulation of myosatellites proliferation and fusion with the maternal fiber.

Data has appeared recently showing that the myosatellite nuclei can incorporate into a fiber under low–intensity chronic training: during low–frequency chronic electro stimulation and voluntary animal activity (“voluntary wheel”) [71]. Such a regime of contractile activity usually does not lead to working hypertrophy. However, myosin phenotype actively shifts to the slow direction. As was shown previously [72], an increase in the slow fiber number during the slow–frequency stimulation of rat fast muscles cannot be explained by the change in the myosin isoforms expression inside a fiber. The suppression of myosatellites multiplication prevented by irradiation was established recently to prevent the transformation of fibers to the slow type during low–frequency chronic stimulation [73]. It is of interest that pharmacological stimulation of PPAR β ( peroxisome proliferator–activated receptor β ) is one of the components of the signaling system switching myosin isoform expression to the slow type in the myonuclei and favors myosatellite fusion with a muscle fiber [74].

Thus, the incorporation of myosatellite nuclei (evidently with a slow pattern of myosin isoform expression) into muscle fiber during prolonged low–frequency stimulation leads to myosin phenotype adaptive changes in the skeletal muscle.

## Myosatellite cells of skeletal muscle during stretch and stretch combined with disuse

Gravitational unloading is a particular type of muscle contractile activity reduction. A sharp decrease in the electric activity of soleus (to the zero level) is observed, as a rule, immediately after support elimination and continues for 2–3 days of disuse. Then electric activity begins its restoration slowly and reaches the control level by the 14th day of real or simulated microgravity [75]. However, gradually increased muscle activity does not prevent muscle atrophy development. Evidently, the decreased contractile activity has an affect alongside with the the significantly declined (to zero under microgravity) resistance to muscle contraction ( weight bearing), which has a significant influence on atrophy development [76]. One approach to studying this factor is chronic or repeated passive stretch of the muscle. This stretch compensates for the lack of gravity loading and certainly prevents the development of muscle regeneration [77].

**Fig. 2:**
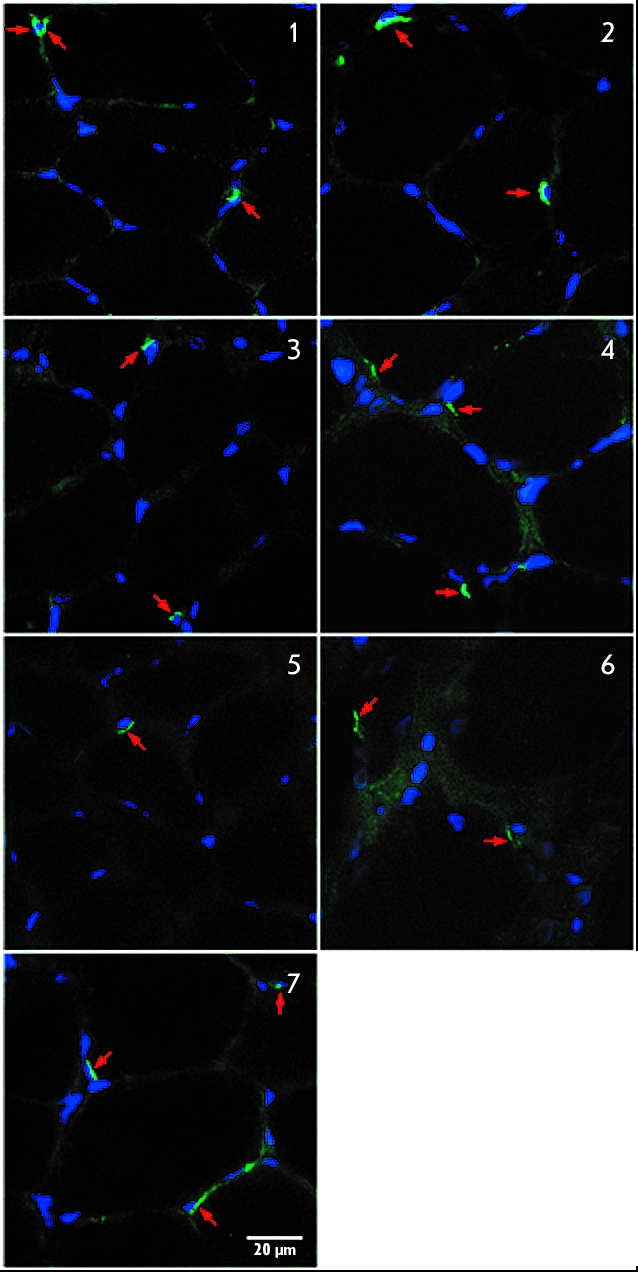
Satellite cells in rat m.soleus transverse section. M-cadherin staining. (1) “Hindlimb suspension 3 days,” (2) “Hindlimb suspension + stretch 3 days,” (3) “Hindlimb suspension 7 days”, (4) “Hindlimb suspension + stretch 7 days”, (5) “Hindlimb suspension 14 days”, (6) “Hindlimb suspension + stretch 14 days”, (7) Control.

The interrelation between stretch and myosatellite activation in culture was demonstrated in the experiments conducted by Tatsumi * et al * . [78]. Resting satellite cells under periodic stretch activated and entered the cell cycle, probably being stimulated by HGF synthesis in the stretched cells. The same authors showed that, during a short stretch (1 h) combined with the hindlimb suspension of rats, mechanical stretch caused nitric oxide (NO) synthesis. The latter induced HGF linked with the muscle fiber surface. HGF binds the c–Met–receptor of the myosatellite cells, leading to their activation. On the other hand, data exist indicating that, during compensatory hypertrophy caused by the synergistic–muscle removal, myonuclei activation can occur independently from the NOS inhibitor [79]. In the model of Wozniak *et al * . [38], isolated muscle stretch, such as during a single fibers stretch, activated myosatellites, which were determined by BrdU incorporation into the dividing cell nuclei. In our study, 3 days of simulated gravitational unloading (hindlimb suspension model) caused no change in the satellite cells expressing m–cadherin in rat soleus, while 7 and 14 days of disuse caused a 30 and 50% decrease in the number of myosatellites, correspondingly, as compared to the control. Passive soleus stretch combined with gravitational unloading made it possible to maintain the amount of satellites 30% higher than in the control at the 3rd and 7th days of disuse, and at the control level until the 14th day of unloading (Fig. 2). We surmised that, after the elimination of the proliferative potential of precursor cells by γ –irradiation, muscle fibers partially lose their ability to maintain fiber size during stretch combined with disuse. A study of local irradiation of a rat shin with a dosage of 2500 rad followed by hindlimb suspension or suspension combined with stretch showed that irradiation did not influence the countermeasure effect of passive stretch (atrophy prevention, fibers transformation, and myonuclei number decrease), which was observed under suspension [80]. Recent * in vivo* experiments demonstrated that L–arginine (NO donor) administration under disuse decreased muscle atrophy and maintained the number of myonuclei and myosatellites at the control level. Moreover, NO–synthetase inhibitor, L–NAME, significantly decreased myosatellite proliferation during stretch combined with animal hindlimb suspension. Thus, one can assume that, in the studied model, NO significantly influences myosatellites proliferation. However, an administration of NO–synthetase blocker did not affect the efficiency of maintaining muscle mass during the stretch (see the significance of myosatellite proliferation during hypertrophy above ) [81].

Myotube stretch in culture leads to the release of other endocrine factors, including IGF. The studies conducted by the Goldspink laboratory [49, 61] showed that, during stretch combined with the electro stimulation of the tibialis anterior and during mechanical injury, myosatellite activation occurred along with IGF–1 mRNA expression. The authors linked the resting myosatellite activation and proliferation during muscle stretch to the expression of MGF (the maximal expression of the splice–variant MGF was observed in the first 4 days after the stretch), while their differentiation and fusion with the muscle fibers depended on the subsequent IGF–1Ea (5–12 days after applying the stretch) [49].

Interestingly, in our studies IGF–1 expression stimulation was observed only on the 7th day of stretch combined with gravitational unloading, while the myosatellite–cell number increased on the 3rd day. The proliferation of these cells during stretch combined with disuse can probably be explained by the earlier MGF expression. Further studies should shed more light on the question. 

 Thus, we can assume that myosatellite proliferation during stretch combined with gravitational unloading, which is stimulated by different mechanisms, is unnecessary for preventing the atrophy of muscle fibers. The myonuclear number is probably maintained in this case due to the antiapoptotic effect of the stretch. However, the concomitant myosatellite activation prevents a decrease in the muscle–regeneration potential.

Therefore, in this review we have discussed one of the most controversial issues surrounding skeletal muscle plasticity: the effect of the contractile activity on the myonuclear pool. The perspectives of pharmacological and gene–therapeutic regulation of myonuclei apoptosis and myosatellite activity are also of importance. NO donors and the recombinant analogues of the growth factors as countermeasures to the atrophy changes in skeletal muscle during posthypokinetic recovery and the rehabilitation of injured athletes need further study.

## ABBREVIATIONS

IGF: insulin-like growth factor
AIF: apoptosis-inducing factor
GFP: green fluorescent protein
BrdU: 5-bromo-2-deoxyuridine
CD34: clusters of differentiation
CD45: clusters of differentiation
CD54: clusters of differentiation
c-Met: HGF receptor
HGF: hepatocyte growth factor
FGF: fibroblast growth factor
MMPs: matrix metalloproteinases
MGF: mechano-growth factor
